# Extensive introgression among strongylocentrotid sea urchins revealed by phylogenomics

**DOI:** 10.1002/ece3.10446

**Published:** 2023-08-25

**Authors:** Matthew R. Glasenapp, Grant H. Pogson

**Affiliations:** ^1^ Department of Ecology and Evolutionary Biology University of California Santa Cruz California USA

**Keywords:** bioinformatics, echinoderms, gamete recognition proteins, hybridization, molecular evolution, phyloinformatics

## Abstract

Gametic isolation is thought to play an important role in the evolution of reproductive isolation in broadcast‐spawning marine invertebrates. However, it is unclear whether gametic isolation commonly evolves early in the speciation process or only accumulates after other reproductive barriers are already in place. It is also unknown whether gametic isolation is an effective barrier to introgression following speciation. Here, we used whole‐genome sequencing data and multiple complementary phylogenomic approaches to test whether the well‐documented gametic incompatibilities among the strongylocentrotid sea urchins have limited introgression. We quantified phylogenetic discordance, inferred reticulate phylogenetic networks, and applied the *Δ* statistic using gene tree topologies reconstructed from multiple sequence alignments of protein‐coding single‐copy orthologs. In addition, we conducted ABBA–BABA tests on genome‐wide single nucleotide variants and reconstructed a phylogeny of mitochondrial genomes. Our results revealed strong mito‐nuclear discordance and considerable nonrandom gene tree discordance that cannot be explained by incomplete lineage sorting alone. Eight of the nine species examined demonstrated a history of introgression with at least one other species or ancestral lineage, indicating that introgression was common during the diversification of the strongylocentrotid urchins. There was strong support for introgression between four extant species pairs (*Strongylocentrotus pallidus* ⇔ *S. droebachiensis*, *S. intermedius* ⇔ *S. pallidus*, *S. purpuratus* ⇔ *S. fragilis*, and *Mesocentrotus franciscanus* ⇔ *Pseudocentrotus depressus*) and additional evidence for introgression on internal branches of the phylogeny. Our results suggest that the existing gametic incompatibilities among the strongylocentrotid urchin species have not been a complete barrier to hybridization and introgression following speciation. Their continued divergence in the face of widespread introgression indicates that other reproductive isolating barriers likely exist and may have been more critical in establishing reproductive isolation early in speciation.

## Graphical abstract

We found widespread evidence for introgression within the strongylocentrotid sea urchin family. The arrows represent strongly supported discrete introgression events uncovered by multiple complementary phylogenomic approaches. Despite well‐documented gametic incompatibilities between species, introgression appears to have been common during the diversification of the strongylocentrotid family.

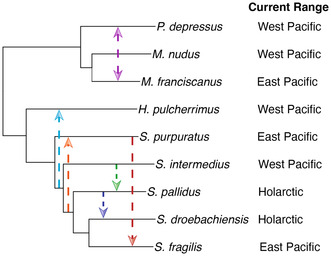

## 1 INTRODUCTION

The new availability of genome‐scale data has stimulated considerable investigation into the genomic architecture of speciation – the number, kind, location, and relative effect size of loci underlying reproductive isolation. Understanding the genetic basis of speciation requires identifying these so‐called “barrier loci” and characterizing the selective agents responsible for their divergence (Orr, 2005). Although it is well established that reproductive isolation often evolves as a by‐product of diversifying selection (Coyne & Orr, 2004), the link between phenotypic divergence and the specific genetic changes underlying reproductive isolation remains weak (Schluter & Rieseberg, 2022). One of the major outstanding questions concerns whether reproductive incompatibilities evolve more commonly from adaptive divergence or nonadaptive processes such as intragenomic conflict and divergent gene duplication resolution (Schluter & Rieseberg, 2022). Contrary to the recent enthusiasm for ecological speciation, hybrid incompatibility loci are often associated with nonadaptive processes (Campbell et al., 2018; Maheshwari & Barbash, 2011; Presgraves, 2010). However, research seeking to identify barrier loci has been historically biased towards postzygotic isolation, which may be less likely to evolve from ecological selection than prezygotic isolation (Campbell et al., 2018). Broader taxonomic representation is needed because most conclusions have been drawn from a limited number of taxa (Campbell et al., 2018).

Studying speciation in the sea offers a unique opportunity to characterize the evolution of reproductive isolation in settings where geographic barriers are less common. Especially compelling are the broadcast‐spawning marine invertebrates, whose life histories and reproductive ecologies differ drastically from most animal speciation models. Broadcast spawners typically have massive fecundities and highly dispersive larvae, resulting in large population sizes and broad geographic ranges. Their high levels of gene flow across large distances and the rarity of absolute geographic barriers should limit opportunities for population differentiation and the evolution of reproductive isolating barriers (Palumbi, 1994). Furthermore, broadcast spawners such as sea urchins lack pre‐mating mechanical and behavioral drivers of reproductive isolation, and incipient species often show little morphological, ecological, or physiological divergence. Despite these constraints, species diversity in broadcast spawners appears high. One explanation for the high species richness observed in the absence of obvious physical barriers and ecological divergence is that the rapid evolution of a small number of reproductive proteins may establish reproductive isolation (Levitan et al., 2019; Metz et al., 1994; Palumbi, 1992, 2009; Palumbi & Metz, 1991; Swanson & Vacquier, 2002b).

Many species of broadcast spawners exhibit species‐specific fertilization mediated by gamete recognition proteins (GRPs) located on the surfaces of sperm and egg cells (Metz et al., 1994; Summers & Hylander, 1975; Vacquier & Moy, 1977). These proteins often evolve rapidly under positive selection and have been implicated in the establishment of reproductive isolation (Biermann, 1998; Lee et al., 1995; Lee & Vacquier, 1992; Metz & Palumbi, 1996; Swanson & Vacquier, 2002a, 2002b; Yang et al., 2000). Furthermore, gametic compatibility among sea urchin species was found to be negatively correlated with sequence divergence of the sperm GRP bindin (Zigler et al., 2005), suggesting that bindin sequence similarity determines gametic compatibility. These discoveries reinforced the hypothesis that speciation in broadcast spawners may occur when diversifying selection at GRPs produces gametic incompatibility, leading to the classification of bindin and its egg receptor protein (EBR1) as speciation genes (Blackman, 2016; Nei & Nozawa, 2011; Noor & Feder, 2006). Several mathematical models have shown that both allopatric and sympatric speciation are theoretically possible when sexual conflict mediated by polyspermy risk drives a coevolutionary chase between the sexes and causes GRP divergence (Gavrilets, 2000; Gavrilets & Hayashi, 2005; Gavrilets & Waxman, 2002; Van Doorn et al., 2001). However, it remains unclear whether divergence in reproductive proteins caused speciation or instead accumulated after significant reproductive isolation had already evolved.

The strongylocentrotid sea urchin family is an ideal group for studying the evolution of reproductive isolation. Due to their translucent embryos, sea urchins became model organisms for fertilization studies during the late 19th century. Like many other marine species, sea urchins have large effective population sizes, broad geographic ranges, and limited population structure. The purple sea urchin, *Strongylocentrotus purpuratus* (Stimpson), is a member of the strongylocentrotid family and has a well‐annotated reference genome in its fifth major revision. It is currently believed that the strongylocentrotid species are strongly reproductively isolated and have not shared alleles through introgression due to well‐documented gametic incompatibilities and the rarity of natural hybrids (Lessios, 2007; Strathmann, 1981). However, recent studies indicate that reproductive isolation may be incomplete, evidenced by introgression between *S. pallidus* (Sars) and *S. droebachiensis* (O. F. Müller) in the Northeast Pacific (Addison & Hart, 2005; Addison & Pogson, 2009; Harper et al., 2007; Pujolar & Pogson, 2011) and Northwest Atlantic (Addison & Hart, 2005; Harper et al., 2007). Whether other strongylocentrotid taxa have experienced introgression remains unknown.

If gametic isolation were an important isolating barrier early in strongylocentrotid speciation events, evidence of introgression should be rare and negatively correlated with phylogenetic distances and gametic incompatibilities. We tested these predictions using whole‐genome sequencing data from the strongylocentrotid urchin species and multiple complementary phylogenomic approaches. Given the documented susceptibility of *S. droebachiensis* eggs to heterospecific sperm (Levitan, 2002b) and the previous finding of *S. pallidus* alleles in *S. droebachiensis* individuals (Addison & Pogson, 2009), we expected to find a signal of introgression between *S. droebachiensis* and other congeners. Further predictions about introgression were challenging for several reasons. First, heterospecific cross data only exists for a few strongylocentrotid species pairs. Second, although fertilization is more efficient in conspecific crosses of strongylocentrotid urchins (Levitan, 2002b; Minor et al., 1991; Strathmann, 1981), heterospecific fertilizations readily occur in no‐choice experiments between highly divergent species (Moore, 1957; Newman, 1923; Zhao et al., 2021). Furthermore, whether hybrid matings occur *naturally* depends heavily upon the distance between a female urchin and the nearest conspecific male (Levitan, 2002b), and little is known about the fitness of hybrid offspring in most heterospecific crosses.

Contrary to our expectation of limited introgression, we found widespread introgression across the strongylocentrotid family at multiple time scales, suggesting that gametic incompatibilities have not been an effective barrier to introgression. The existing gametic incompatibilities either were not strong enough to prevent significant introgression or evolved after significant introgression had already occurred, both of which are inconsistent with gametic isolation establishing reproductive isolation and causing speciation. Our findings indicate that additional reproductive barriers must have been in place for the establishment and maintenance of species barriers.

## 2 MATERIALS AND METHODS

### 2.1 Study system

The strongylocentrotid phylogeny comprises two major clades: Clade S includes *Strongylocentrotus* and *Hemicentrotus*; Clade M includes *Mesocentrotus* and *Pseudocentrotus*. Both *Hemicentrotus* and *Pseudocentrotus* are monotypic genera. The phylogeny is parsimoniously consistent with a Western Pacific common ancestor and at least two independent Eastern Pacific colonizations (Kober & Bernardi, 2013). Four species are limited to the Northwest Pacific: *P. depressus* (A. Agassiz), *M. nudus* (A. Agassiz), *H. pulcherrimus* (A. Agassiz), and *S. intermedius* (A. Agassiz). An additional two species, *S. pallidus* and *S. droebachiensis*, are found in, but not limited to, the Northwest Pacific. Five species co‐occur in the East Pacific with overlapping geographic ranges, depth preferences, and spawning seasons: *S. droebachiensis*, *S. fragilis* (Jackson), *S. pallidus*, *S. purpuratus*, and *M. franciscanus* (A. Agassiz). *S. droebachiensis* and *S. pallidus* have further expanded their ranges, crossing the Bering Sea to colonize the Arctic Ocean and the West and East Atlantic. These two species show little differentiation between the Pacific and Atlantic Oceans, likely due to stepping‐stone populations that facilitate gene flow (Palumbi & Kessing, 1991).

### 2.2 Whole‐genome resequencing and data pre‐processing

The genomes of all strongylocentrotid species had been previously sequenced at high coverage depth with the Illumina HiSeq 2500 (Kober & Bernardi, 2013; Kober & Pogson, 2017). The raw sequencing reads were deposited in the NCBI Sequence Read Archive under BioProject PRJNA391452. Metadata for the genome samples is available in Table S1. The sequencing reads were pre‐processed with Picard (Broad Institute, 2018) and GATK v4.2.6.1 following GATK's Best Practices (Van der Auwera et al., 2013). Adapter sequences were marked using Picard MarkIlluminaAdapters, sequencing reads were mapped to the *S. purpuratus* reference genome (Spur_5.0) using bwa‐mem2 v2.2.1 (Vasimuddin et al., 2019), and duplicate reads were marked with Picard MarkDuplicates. Reference mapping and alignment were evaluated using samtools flagstat (Danecek et al., 2021) and mosdepth v0.3.3 (Pedersen & Quinlan, 2018).

Variant calling and joint genotyping were performed using GATK's HaplotypeCaller and GenotypeGVCFs. Variant quality filtering was performed independently for each subset of species used in downstream analyses. Vcf files were hard‐filtered for variants with skewed values across all samples following GATK recommendations. Single nucleotide variants (SNVs) were filtered that had low quality (QUAL < 30), low map quality (MQ < 40), low quality by depth scores (QD < 2), high Fisher strand scores (FS > 60), high strand odds ratios (SOR > 3), low mapping quality rank sum scores (MQRankSum < −12.5), or low read position rank sum scores (ReadPosRankSum < −8). Indels were filtered that had low quality (QUAL < 30), low quality by depth scores (QD < 2), high Fisher strand scores (FS > 200), or low read position rank sum scores (ReadPosRankSum < −20.0). Furthermore, individual genotypes with low quality (GQ < 20) or low read depth (DP < 3) were set to missing, and SNVs within three base pairs of an indel were filtered.

### 2.3 Phylogenetic relationships and concordance factor statistics

For phylogenetic inference, multiple sequence alignments were created for protein‐coding single‐copy orthologs inferred by filtering *S. purpuratus* nuclear gene models by coverage depth. Genes were filtered if any sample had a mean depth lower than 10×, a mean depth greater than double the sample's mean depth for *S. purpuratus* exons, or fewer than 75% of the bases in the gene covered by 10 reads. To account for nonindependence among loci, genes were filtered so that there was a minimum of 20 kb between included loci. Multiple sequence alignments of concatenated CDS were created for each gene passing filter by applying the hard‐filtered SNVs and deletions to the *S. purpuratus* reference sequence using vcf2fasta (Sanchez‐Ramirez, 2017). Insertions were ignored to keep gene coordinates consistent with the *S. purpuratus* reference. After creating the fasta alignments, genes were excluded if they had no parsimony informative sites or if their length was not a multiple of three.

A maximum likelihood species tree was inferred using the edge‐linked partition model of IQ‐TREE (Chernomor et al., 2016; Nguyen et al., 2015) on the concatenated single‐copy ortholog fasta alignments. Branch supports were obtained using ultrafast bootstrap with 1000 replicates (Hoang et al., 2018). Single locus trees were reconstructed for each single‐copy ortholog fasta alignment using IQ‐TREE's ModelFinder (Kalyaanamoorthy et al., 2017).

Gene concordance factor (gCF) and site concordance factor (sCF) statistics (Minh et al., 2020) were calculated for each branch in the species tree to quantify the amount of phylogenetic discordance present in the data. For each branch in the species tree, the gCF measures the proportion of gene trees containing that branch, while the sCF measures the proportion of informative sites concordant with that branch. The sCFs were calculated by randomly sampling 300 quartets around each internal branch in the phylogeny using an updated version of sCF based on maximum likelihood implemented in IQ‐TREE v2.2.2 (Mo et al., 2022). In addition to the gCF and sCF values, IQ‐TREE also calculates the frequencies of the two discordant trees produced by nearest‐neighbor interchanges (NNI) around each branch. Coalescent theory predicts that the two discordant trees should be equally observed if the discordance is caused by incomplete lineage sorting (ILS) only. However, one tree may become more frequent than the other if introgression has occurred. To test for introgression, chi‐square tests were used to compare counts of the two discordant NNI trees for each branch in the species tree.

### 2.4 Mitochondrial phylogenetics

To investigate the relationships between mitochondrial genomes and look for signs of introgression, mitochondrial genomes were assembled for the same samples used in the species tree inference (Kober & Bernardi, 2013; Kober & Pogson, 2017). Metadata for the mitochondrial genomes is available in Table S1. The *S. purpuratus* sample used was from the original reference genome assembly (NC_001453.1; Jacobs et al., 1988). The sequences were aligned with Clustal Omega v1.2.3 (Sievers et al., 2011; Sievers & Higgins, 2018), and a maximum likelihood tree was created with IQ‐TREE using ModelFinder. Branch supports were obtained using ultrafast bootstrap with 10,000 replicates.

### 2.5 Tests for introgression

Recent powerful phylogenomic approaches for characterizing introgression based on the multi‐species coalescent (MSC) model make it possible to detect introgression with just a single genome sample per species (Hibbins & Hahn, 2022). Due to limited a priori hypotheses about which species may have experienced introgression, we implemented several independent tests for introgression based on gene tree discordance that uses different inference methods. Patterson's *D* statistic uses genome‐wide counts of biallelic site patterns (Durand et al., 2011; Green et al., 2010), the *Δ* statistic uses genome‐wide counts of gene genealogies (Huson et al., 2005), and PhyloNet uses maximum likelihood to estimate reticulate phylogenies using distributions of gene genealogies (Nguyen et al., 2015; Than et al., 2008).

#### 2.5.1 Patterson's *D* statistic

Patterson's *D* statistic, or the ABBA–BABA test, is the most widely used summary statistic in introgression studies and is robust in a wide parameter space (Kong & Kubatko, 2021; Zheng & Janke, 2018). Patterson's *D* statistic tests for a genome‐wide imbalance in the counts of the biallelic site patterns consistent with the two possible discordant topologies in a rooted triplet (Durand et al., 2011; Green et al., 2010). Significance for *D* is calculated using a block jackknife approach that accounts for nonindependence among sites in the data. Patterson's *D* statistic was calculated for all phylogenetically relevant triplets using the genome‐wide genotype call set and the Dsuite Dtrios program (Malinsky et al., 2021) with a block‐jackknife size of 1 Mb. For comparisons within the S clade, separate tests were run with *M. nudus*, *M. franciscanus*, and *P. depressus* as outgroups. For the test within the M clade, *S. purpuratus* and *S. fragilis* were used as the outgroup. A recent addition to Patterson's *D*, *D*_p_, can approximate the genome‐wide introgression proportion (Hamlin et al., 2020) and was calculated for each triplet using the Dsuite output. To determine whether introgression is correlated with phylogenetic distance or GRP divergence, we performed linear regressions of mean Patterson's *D* and *D*_p_ by overall phylogenetic distance, bindin distance, and EBR1 distance (Appendix S1).

#### 2.5.2 *Δ* statistic

The *Δ* statistic is an alternative approach to Patterson's *D* that uses counts of discordant gene tree topologies rather than site patterns (Huson et al., 2005). *Δ* is less sensitive to the assumption of Patterson's *D* that there have not been multiple substitutions per site (Hahn, 2018) and was used as a secondary measure to confirm significant Patterson's *D* statistic tests where introgression must have occurred between extant taxa. *Δ* was estimated using gene tree topologies reconstructed from multiple sequence alignments of single‐copy orthologs for three different quartets: (((*M. nudus*, *M. franciscanus*), *P. depressus*), *S. purpuratus*); (((*S. droebachiensis*, *S. pallidus*), *S. intermedius*), *M. franciscanus*); (((*S. fragilis*, *S. droebachiensis*), *S. pallidus*), *M. franciscanus*). Significance was assessed by calculating *Δ* for 10,000 pseudoreplicate datasets created by resampling the gene tree topologies with replacement (Vanderpool et al., 2020).

#### 2.5.3 PhyloNet

The PhyloNet software package implements a powerful set of likelihood methods based on the multispecies network coalescent (MSNC) model (Meng & Kubatko, 2009) that can be used to formally test for introgression (Than et al., 2008; Wen et al., 2018). PhyloNet programs can identify introgression on the internal branches of a phylogeny and reliably infer the direction of introgression (Hibbins & Hahn, 2022). To further characterize the history of introgression within the strongylocentrotid family, we ran PhyloNet's InferNetwork_ML program (Yu et al., 2014) with reconstructed gene tree topologies to infer phylogenetic networks with reticulation edges representing discrete introgression events. A smaller subset of species was used in the PhyloNet analysis due to computational constraints and the requirement that the gene trees be rooted. A new set of single‐copy orthologs was inferred for *M. franciscanus*, *H. pulcherrimus*, and the five *Strongylocentrotus* taxa (Table S10). Gene trees were estimated with IQ‐TREE2, and 100 bootstrap trees were generated for each gene using standard nonparametric bootstrap to account for uncertainty in gene tree reconstruction. InferNetwork_ML was run to infer phylogenetic networks with 0, 1, 2, and 3 reticulations.

## 3 RESULTS

### 3.1 Data pre‐processing

The results of the reference genome mapping are summarized in Table 1. The read mapping percentage per sample ranged from 76% to 98%. Mean genome‐wide coverage depth typically ranged from 18× to 32×, except for *S. purpuratus* and *S. pallidus*. Coverage depth for *S. pallidus* (12×) was lower because of a reduced library complexity resulting from the early developmental phase of automated library preparation protocols (Kober & Pogson, 2017). *S. purpuratus* was sequenced at a higher depth (91×) for reference genome assembly. Mean coverage depth increased to >38× for protein‐coding single‐copy orthologs, except for *S. pallidus* (15×). Additional coverage metrics are presented in Tables S3–S5.

**TABLE 1:** Summary of genomic DNA sequencing, reference mapping, and coverage.

Species	Reference mapping			% Bases covered			Mean coverage depth		
	Raw reads	Mapped %	Proper pair %	Whole genome^a^ (%)	Coding^b^ (%)	Single‐copy orthologs 10×^c^	Whole genome^d^	Coding^e^	Single‐copy orthologs^f^
Sdro	3.04E+08	91.74	78.11	78	92	0.97	24.7×	41.5×	42.5×
Sfra	3.97E+08	89.87	78.21	81	93	0.97	32.1×	46.8×	48.2×
Spal	1.50E+08	91.82	72.39	78	91	0.97	11.9×	15×	15.5×
Sint	4.01E+08	84.24	73.06	77	91	0.97	28.3×	44.2×	50.3×
Spur	6.21E+08	98.11	89.04	99	100	0.99	91.3×	100.3×	108.2×
Hpul	3.76E+08	82.71	68.67	69	86	0.95	24.5×	44.3×	53.3×
Mnud	3.82E+08	77.00	63.08	58	82	0.92	21.1×	40.5×	45.3×
Mfra	3.39E+08	80.36	64.30	60	84	0.93	19.9×	33.8×	38.3×
Pdep	3.28E+08	76.17	60.79	50	77	0.89	18.1×	47.5×	53.5×

Species abbreviations: *Sdro, S. droebachiensis*; *Sfra, S. fragilis*; *Spal, S. pallidus*; *Sint*, *S. intermedius*; *Spur, S. purpuratus*; *Hpul, H. pulcherrimus*; *Mnud, M. nudus*; *Mfra, M. franciscanus*; *Pdep, P. depressus*.

^a^ Percentage of bases in the *S. purpuratus* reference genome covered by at least one read.

^b^ Percentage of coding bases in the *S. purpuratus* reference genome covered by at least one read.

^c^ Percentage of single‐copy ortholog coding bases covered at 10× depth.

^d^ Mean genome‐wide coverage depth of the *S. purpuratus* reference genome.

^e^ Mean coverage depth for 246,202 unique exons in the *S. purpuratus* genome assembly.

^f^ Mean coverage depth of coding bases for 4497 single‐copy orthologs.

### 3.2 Phylogenetic discordance among strongylocentrotids

**FIGURE 1:**
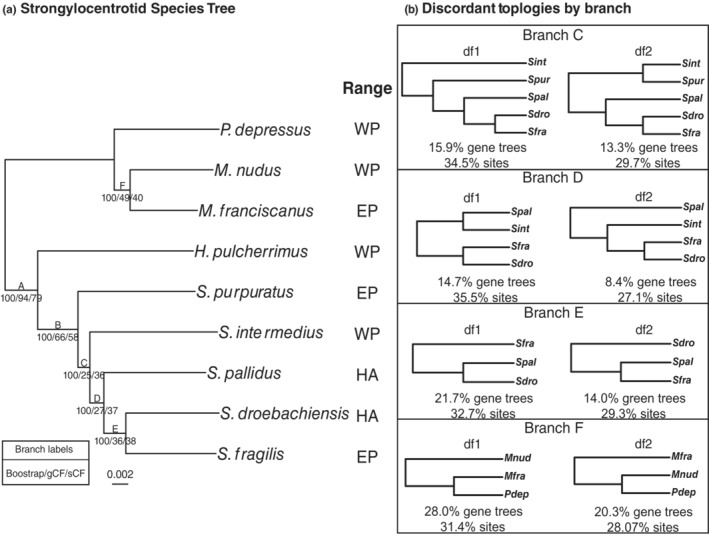
(a) Phylogeny of the nine strongylocentrotid sea urchin species included in the study. A maximum likelihood species tree was inferred using the edge‐linked partition model of IQ‐TREE (Chernomor et al., 2016; Nguyen et al., 2015) on 4497 concatenated single‐copy ortholog alignments. Branch supports were obtained using ultrafast bootstrap (Hoang et al., 2018) with 1000 replicates. Gene concordance factor (gCF) and site concordance factor (sCF) statistics (Minh et al., 2020; Mo et al., 2022) were calculated using IQ‐TREEv2.2.2. For each branch in the species tree, the gCF measures the proportion of gene trees containing that branch, while the sCF measures the proportion of informative sites concordant with that branch (Minh et al., 2020). (b) Extended output from the gene concordance factor statistics, showing the most frequent discordant topologies (df1, df2) for branches in the species tree with significant imbalances in the frequencies of df1 and df2. The frequencies of the df1 and df2 topologies are expected to be equal under incomplete lineage sorting alone. Species abbreviations: *Sdro, S. droebachiensis*; *Sfra, S. fragilis*; *Spal, S. pallidus*; *Sint*, *S. intermedius*; *Spur, S. purpuratus*; *Hpul, H. pulcherrimus*; *Mnud, M. nudus*; *Mfra, M. franciscanus*; *Pdep, P. depressus*.

Although the inferred maximum likelihood species tree topology agreed with the topology produced by Kober and Bernardi (2013), the gene and site concordance factor statistics revealed extensive phylogenetic discordance on most species tree branches (Figure 1a, Table S6). The three internal branches relating the *Strongylocentrotus* species had very low gCF and sCF values. These branches are short, and the lower gCF values than sCF values signal that error in gene tree reconstruction likely contributed to the observed signal of phylogenetic discordance. However, the low sCF values suggest that there is not overwhelming support for any single resolution of these branches, implying considerable ILS or introgression. Although the low gCF values may be partially explained by error in gene tree reconstruction, biases in the frequencies of the discordant topologies are suggestive of introgression (Figure 1b, Table S6). For the branch in the species tree placing *S. purpuratus* as the outgroup to the rest of the *Strongylocentrotus* species (Branch C), the discordant resolution placing *S. intermedius* as the first diverging member of *Strongylocentrotus* (15.9% gene trees, 34.5% sites) was observed more frequently than the other NNI discordant resolution (13.3% gene trees, 29.7% sites, *p* = .0015), indicating introgression between *S. purpuratus* and one or more of *S. pallidus*, *S. droebachiensis*, *S. fragilis*, or an ancestral lineage. Three other branches also had a discordant topology that was significantly overrepresented (Branches D, E, F), implying introgression between *S. intermedius* ⇔ *S. pallidus*, *S. pallidus* ⇔ *S. droebachiensis*, and *P. depressus* ⇔ *M. franciscanus* (Figure 1b).

### 3.3 Mitochondrial introgression

**FIGURE 2:**
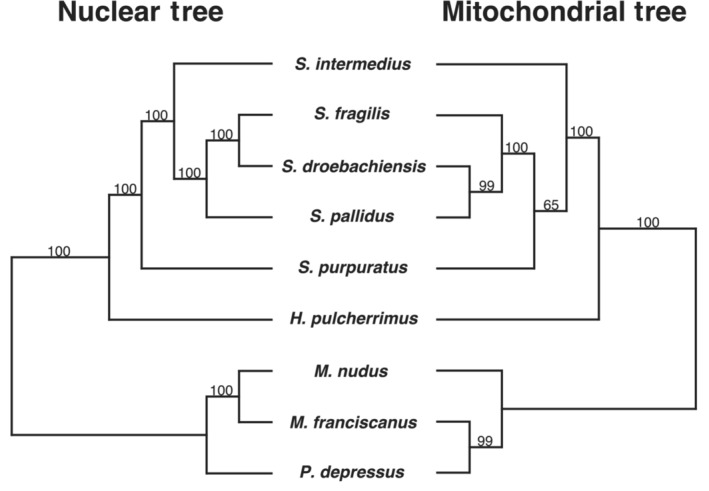
A maximum likelihood tree of mitochondrial genome assemblies was inferred from the same samples used in the nuclear species tree shown in Figure 1a. Both nuclear and mitochondrial trees were rooted at the midpoint. The mitochondrial genomes were aligned using Clustal Omega v1.2.3, and a maximum likelihood tree was constructed using IQ‐TREE (Nguyen et al., 2015) and ModelFinder (Kalyaanamoorthy et al., 2017). Branch supports were obtained using ultrafast bootstrap (Hoang et al., 2018) with 1000 replicates. Relative to the true species relationships (Figure 1a), the placements of the following are swapped: (i) *M. nudus* and *P. depressus*, (ii) *S. purpuratus* and *S. intermedius*, and (iii) *S. pallidus* and *S. fragilis*.

The phylogeny of the mitochondrial genome accessions did not recover the true species relationships, showing several discordant patterns consistent with introgression (Figure 2). *M. franciscanus* clustered with *P. depressus* with 99 percent bootstrap support rather than with its sister taxon, *M. nudus*. Similarly, *S. droebachiensis* clustered with *S. pallidus* with 99% bootstrap support rather than its sister taxon, *S. fragilis*. The last source of discordance was the placement of *S. purpuratus* and *S. intermedius*. In the mitochondrial tree, the positions of *S. purpuratus* and *S. intermedius* are swapped relative to the species tree, consistent with gene flow between *S. purpuratus* and one or more of *S. pallidus*, *S. droebachiensis*, *S. fragilis*, or an ancestral lineage. All three of these discordant topologies were also overrepresented in the gene concordance factor analysis, indicating that the mito‐nuclear discordance observed was caused by introgression.

### 3.4 Introgression tests

#### 3.4.1 Patterson's *D* statistic

**FIGURE 3:**
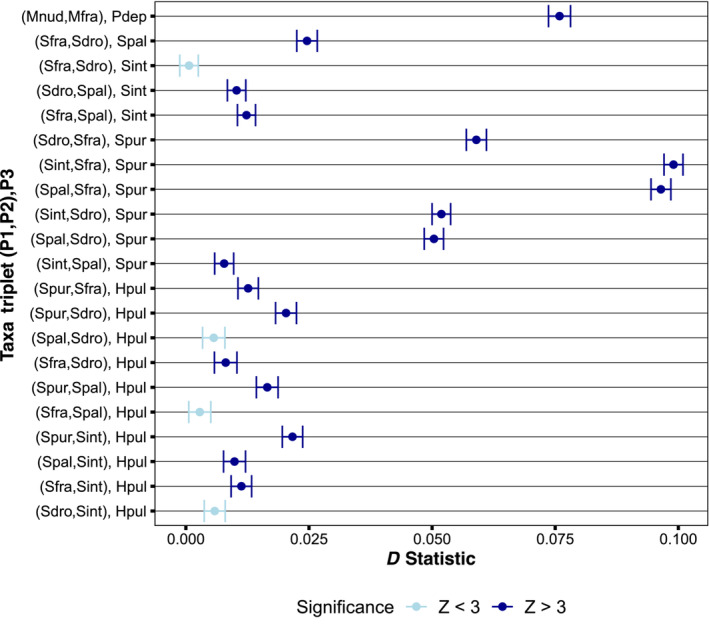
Results of ABBA–BABA tests for all phylogenetically relevant triplets. Equal numbers of ABBA and BABA sites are expected under the null hypothesis of no introgression (*D* = 0). A positive *D* statistic indicates introgression between P3 and P2. Significance was assessed using a block jackknife size of 1 Mb. Error bars represent the standard error. Species abbreviations: *Sdro, S. droebachiensis*; *Sfra, S. fragilis*; *Spal, S. pallidus*; *Sint, S. intermedius*; *Spur, S. purpuratus*; *Hpul, H. pulcherrimus*; *Mnud, M. nudus*; *Mfra, M. franciscanus*; *Pdep, P. depressus*.

Seventeen of the 21 Patterson's *D* tests were significant, implicating 10 independent species pairs in introgression (Figure 3, Table 2). For simplicity, only the results with *M. nudus* and *S. purpuratus* as the outgroup are displayed (Figure 3, Table 2). However, the results were consistent regardless of the outgroup choice, and the full results are provided in Tables S7–S9. In the M clade, there was support for introgression between *P. depressus* and *M. franciscanus*. In the S clade, there was evidence for introgression between *H. pulcherrimus* and each of *S. intermedius*, *S. pallidus*, *S. droebachiensis*, and *S. fragilis*. There was also support for introgression between *S. purpuratus* and each of *S. pallidus*, *S. fragilis*, and *S. droebachiensis*. Two additional species pairs were implicated in introgression: *S. intermedius* and *S. pallidus*, and *S. pallidus* and *S. droebachiensis*. In cases where a taxon shows introgression with several species that form a monophyletic group, it may be more parsimonious to assume that introgression occurred between that taxon and the MRCA of the monophyletic group, an internal branch in the phylogeny (Suvorov et al., 2022). For example, it is likely that *H. pulcherrimus* experienced introgression with the common ancestor of the four youngest *Strongylocentrotus* taxa rather than with each of them independently. Similarly, the significant tests involving *S. purpuratus* could have been produced by a single introgression event between *S. purpuratus* and the MRCA of *S. pallidus*, *S. droebachiensis*, and *S. fragilis*. This would reduce the total number of introgression events from 10 to 5, a conservative number because introgression could have occurred both on the internal and terminal branches.

**TABLE 2:** Results of ABBA–BABA tests with Dsuite. The tests are organized by P3 taxon. Equal numbers of ABBA and BABA sites are expected under the null hypothesis of no introgression (*D* = 0). A positive *D* statistic indicates introgression between P3 and P2. Significance was assessed using a block jackknife size of 1 Mb. The *D*_p_ statistic estimates the proportion of the genome supporting introgressed ancestry.

Samples			Dsuite						
P1	P2	P3	*D*	*Z*	*p*	*D*_p_	BBAA	ABBA	BABA
Mnud	Mfra	Pdep	0.076	33.8	.000	0.040	240,218	144,747	124,331
Sfra	Sdro	Spal	0.025	11.8	.000	0.013	319,896	185,499	176,591
Sfra	Sdro	Sint	0.001	0.3	.735	0.000	427,693	185,058	184,824
Sdro	Spal	Sint	0.010	5.5	.000	0.006	249,986	187,513	183,693
Sfra	Spal	Sint	0.012	6.7	.000	0.007	250,248	194,472	189,743
Sdro	Sfra	Spur	0.059	28.9	.000	0.026	490,027	200,788	178,420
Sint	Sfra	Spur	0.099	51.5	.000	0.062	289,884	271,623	222,678
Spal	Sfra	Spur	0.096	47.9	.000	0.055	292,707	210,001	173,050
Sint	Sdro	Spur	0.052	27.5	.000	0.032	278,541	239,301	215,697
Spal	Sdro	Spur	0.050	25.7	.000	0.028	297,221	189,217	171,072
Sint	Spal	Spur	0.008	4.0	.000	0.005	251,450	194,590	191,590
Spur	Sfra	Hpul	0.013	6.1	.000	0.005	443,234	162,520	158,463
Spur	Sdro	Hpul	0.020	9.6	.000	0.009	406,457	159,147	152,805
Spal	Sdro	Hpul	0.006	2.5	.013	0.002	411,339	115,830	114,528
Sfra	Sdro	Hpul	0.008	3.5	.000	0.002	608,640	119,046	117,138
Spur	Spal	Hpul	0.017	7.5	.000	0.007	342,870	139,011	134,494
Sfra	Spal	Hpul	0.003	1.3	.206	0.001	414,614	118,974	118,304
Spur	Sint	Hpul	0.022	10.5	.000	0.010	406,767	172,255	164,957
Spal	Sint	Hpul	0.010	4.4	.000	0.004	370,005	128,140	125,634
Sfra	Sint	Hpul	0.011	5.4	.000	0.005	436,461	156,898	153,403
Sdro	Sint	Hpul	0.006	2.8	.006	0.002	417,256	149,052	147,317

Species abbreviations: *Sfra, S. fragilis*; *Sdro, S. droebachiensis*; *Spal, S. pallidus*; *Sint, S. intermedius*; *Spur, S. purpuratus*; *Hpul, H. pulcherrimus*; *Mnud, M. nudus*; *Mfra, M. franciscanus*; *Pdep, P. depressus*.

We found no significant correlations between Patterson's *D* and overall phylogenetic distance, bindin distance, and EBR1 distance (Appendix S1). Furthermore, when only including *Strongylocentrotus* species, we found a significant, positive correlation between introgression (Patterson's *D*, *D*_p_) and overall phylogenetic distance. The two *Strongyloentrotus* species pairs with the highest overall phylogenetic distances also had the highest mean values of Patterson's *D* and *D*_p_ (*S. purpuratus* – *S. fragilis*, *S. purpuratus* – *S. droebachiensis*).

#### 3.4.2 *Δ* statistic

*Δ* was significantly positive for each of the three quartets tested, signaling introgression between *P. depressus* and *M. franciscanus*, *S. intermedius* and *S. pallidus*, and *S. pallidus* and *S. droebachiensis* (Table 3). All three test results were consistent with the estimated Patterson's *D* statistics (Figure 3, Table 2).

**TABLE 3:** Results of *Δ* analysis.

Samples	*Δ* Analysis						
Quartet	Trees^a^	Concordant^b^	Discordant 1^c^	Discordant 2^d^	*Δ*	SE	Z
(((Sfra,Sdro),Spal),Mfra)	2085	974	639	472	0.15	0.03	5.04
(((Sdro,Spal,),Sint),Mfra)	2107	1104	550	453	0.10	0.03	3.06
(((Mnud,Mfra),Pdep),Spur)	2416	1187	683	546	0.11	0.03	3.94

Species abbreviations: *Sdro, S. droebachiensis*; *Sfra, S. fragilis*; *Spal, S. pallidus*; *Sint, S. intermedius*; *Spur, S. purpuratus*; *Mnud, M. nudus*; *Mfra, M. franciscanus*; *Pdep, P. depressus*.

^a^ Total number of gene trees reconstructed from single‐copy orthologs.

^b^ Number of gene trees that were concordant with the species tree relationships (((P1,P2),P3),O).

^c^ Number of gene trees that had the discordant relationship (((P2,P3),P1),O).

^d^ Number of gene trees that had the discordant relationship (((P1,P3),P2),O).

#### 3.4.3 PhyloNet

**FIGURE 4:**
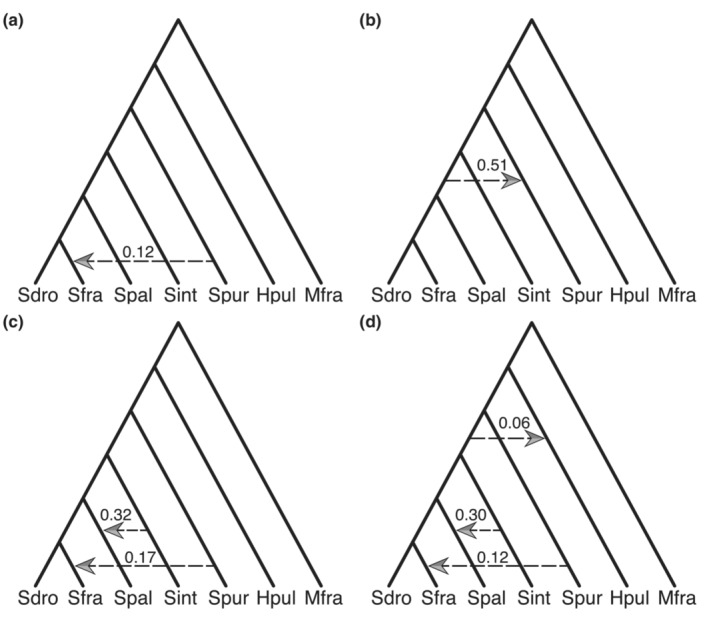
Phylogenetic networks with reticulation edges and inheritance probabilities inferred by PhyloNet InferNetwork_ML. The inheritance probabilities represent the proportion of sampled genes inherited through gene flow. The network with zero reticulation edges recovered the species relationships and had a log‐likelihood of −11,054 (not shown). (a) The best network with one reticulation edge (log‐likelihood: −10,966). (b) The second‐best network with one reticulation edge (log‐likelihood: −10,976). (c) The network inferred with two reticulation edges (log likelihood: −10,929). (d) The network inferred with three reticulation edges (log‐likelihood: −10,903). Species abbreviations: *Sdro, S. droebachiensis*; *Sfra, S. fragilis*; *Spal, S. pallidus*; *Sint, S. intermedius*; *Spur, S. purpuratus*; *Hpul, H. pulcherrimus*; *Mfra, M. franciscanus*.

The PhyloNet analysis revealed similar patterns of introgression to the Patterson's *D* and *Δ* statistics. Conditioning on the species tree backbone, the one‐reticulation edge phylogenetic network with the highest likelihood implied introgression from *S. purpuratus* into *S. fragilis* (Figure 4a). The *D* statistic with the highest magnitude also demonstrated introgression between *S. purpuratus* and *S. fragilis* (Figure 3, Table 2). The network with the next highest likelihood implied introgression between *S. purpuratus* and the *S. droebachiensis* – *S. fragilis* – *S. pallidus* MRCA (Figure 4b), consistent with the gene concordance factor analysis and mitochondrial phylogeny. The best network with two reticulation edges had an additional edge implying introgression from *S. intermedius* into *S. pallidus* (Figure 4c), and the network with three reticulation edges added a third edge indicating introgression from the MRCA of *S. intermedius, S. pallidus*, *S. droebachiensis*, and *S. fragilis* into *H. pulcherrimus* (Figure 4d).

## 4 DISCUSSION

### 4.1 Widespread introgression among the strongylocentrotid urchins

Our study is the first to describe genome‐wide patterns of introgression among sea urchins. It is currently believed that only limited introgression has occurred among sea urchins, but the results of our study indicate that it may be common, at least within *Strongylocentrotidae*. The ubiquity of introgression among the strongylocentrotid taxa suggests that gametic isolation has not been an effective barrier to introgression and may not have played a major role in speciation.

Our tests for introgression revealed that eight out of the nine species included in the study experienced introgression with at least one other species or ancestral lineage. The introgression patterns are clear and consistent regardless of the methodology used (Table 4). A minimum of six introgression events is supported by the data and is a conservative estimate for several reasons. First, we collapsed all tests where a species showed introgression with multiple species forming a monophyletic group. Second, it was not possible to test for introgression between the two pairs of sister taxa as methods relying on phylogenetic discordance cannot detect introgression between sister taxa. Third, we could not rule out introgression in the one species that did not show introgression (*M. nudus*) because the only taxa triplet we could test in the M clade, ((*M. nudus*, *M. franciscanus*), *P. depressus*), implied significant introgression between *P. depressus* and *M. franciscanus*. Finally, we could not test for introgression between the M and S clade members without high‐quality sequence data from a close outgroup to the family. We stress that these are historical introgression events in which the genomic signal has been preserved for millions of years in most cases. Given (i) the methods employed here test for ancient introgression, (ii) introgression is likely not ongoing in most cases, and (iii) only a single diploid genome per species was sampled, we find it likely that the observed signal of introgression was driven by introgressed variation that has been fixed. Furthermore, given that population structure is nearly non‐existent in these sea urchin species (Palumbi & Kessing, 1991; Palumbi & Wilson, 1990), it is likely that most populations and individuals of introgressed taxa would show a similar signal of introgressed ancestry.

**TABLE 4:** Summary of the phylogenomic methods supporting different introgression events.

Taxa	Analysis				
	gCF/sCF	mtDNA	Patterson's *D*	*Δ*	PhyloNet
	Input data				
	4497 Single‐copy orthologs	Mitochondrial genome assemblies	Genome‐wide SNVs	Single‐copy orthologs^a^	2224 Single‐copy orthologs
Mfra – Pdep	×	×	×	×	nt
Spal – Sdro	×	×	×	×	
Sint – Sdro				nt	
Sint – Spal	×		×	×	×
Spur – Sfra			×	nt	×
Spur – Sdro			×	nt	
Spur – Spal			×	nt	
Hpul – Sfra			×	nt	
Hpul – Sdro			×	nt	
Hpul – Spal			×	nt	
Hpul – Sint			×	nt	
Hpul – Sdro/Spal/Sfra/Sint MRCA			×	nt	×
Spur – Sdro/Sfra/Spal MRCA	×	×	×	nt	×

Abbreviations: nt, not tested; SNVs, single nucleotide variants.

Species abbreviations: *Sdro*, *S. droebachiensis; Sfra, S. fragilis; Spal, S. pallidus; Sint, S. intermedius; Spur, S. purpuratus; Hpul, H. pulcherrimus; Mnud, M. nudus; Mfra, M. franciscanus; Pdep, P. depressus*.

^a^ The number of single‐copy orthologs varied depending on the taxa triplet tested. See Table 3 for counts.

Despite considerable phylogenetic discordance in the underlying data, there was strong support for all branches in the strongylocentrotid species tree. This is unsurprising given that these species are well‐diverged, with the youngest pair of sister taxa evolving 4–6 million years ago (Kober & Bernardi, 2013). Incomplete lineage sorting is expected to be pervasive in species with high levels of polymorphism, and the five *Strongylocentrotus* taxa speciated relatively rapidly 4–9 mya (Kober & Bernardi, 2013), resulting in short internal branches. However, incomplete lineage sorting alone is insufficient to explain the observed discordance patterns.

The *D*, *Δ*, and gCF/sCF statistics implied introgression between at least three pairs of extant taxa: *S. pallidus* ⇔ *S. droebachiensis*, *S. intermedius* ⇔ *S. pallidus*, and *P. depressus* ⇔ *M. franciscanus*. Introgression between *S. purpuratus* and *S. fragilis* also likely occurred, but the signal could also be explained by introgression on an internal branch. The mitochondrial phylogeny supported two of these introgression events (*S. pallidus* ⇔ *S. droebachiensis, P. depressus* ⇔ *M. franciscanus*), and the PhyloNet analysis supported introgression between *S. intermedius* and *S. pallidus*, and *S. purpuratus* and *S. fragilis*.

Due to limitations in the fossil record, little is known about the geography of strongylocentrotid urchin speciation and the historical ranges of its extant taxa. However, the patterns of introgression help fill in some of these gaps by demonstrating that some currently allopatric species showing signals of introgression must have had overlapping ranges in the past. For example, the strong signal of introgression between *P. depressus* and *M. franciscanus* was unexpected, given that the ranges of these two species are currently separated by an ocean basin. The M clade phylogeny of the strongylocentrotid family is consistent with a West Pacific common ancestor (Kober & Bernardi, 2013), followed by the colonization of the East Pacific by *M. franciscanus*. Therefore, introgression must have occurred at a time of range overlap in the distant past, implying that *M. franciscanus* speciated in the West Pacific, interbred with sympatric *P. depressus* before colonizing the East Pacific, and later became locally extinct in the West Pacific.

It was similarly unexpected to find support for introgression between *S. intermedius* and *S. pallidus*, given their current distributions. Although *S. intermedius* and *S. pallidus* co‐occur in the Sea of Japan, the *S. pallidus* sample used in this study was from coastal Washington State, indicating that the signal of introgression is ancient. The net direction of gene flow inferred by PhyloNet was from *S. intermedius* into *S. pallidus*, implying that introgression must have occurred before *S. pallidus* expanded its range into the East Pacific. Whether introgression is ongoing between *S. intermedius* and *S. pallidus* in the Sea of Japan is unknown.

Introgression also likely occurred between extant taxa and ancestral lineages (i.e., internal branches). While the optimal phylogenetic network with one reticulation edge implied introgression from *S. purpuratus* into *S. fragilis*, a second network with a similar likelihood supported introgression from the *S. droebachiensis* – *S. fragilis – S. pallidus* MRCA into *S. purpuratus*. Both networks are consistent with the Patterson's *D* statistic results as there was support for introgression between *S. purpuratus* and each of *S. droebachiensis*, *S. fragilis*, and *S. pallidus*. Both the mitochondrial phylogeny and the concordance factor analysis were also consistent with introgression on an internal branch. In the mitochondrial phylogeny, *S. purpuratus* is pulled down as a sister to the *S. droebachiensis* – *S. fragilis* – *S. pallidus* MRCA and the concordance factor analysis revealed that this topology was overrepresented. A similar potential case of introgression on an internal branch was evidenced by the optimal phylogenetic network with three reticulation edges, which implied introgression between *H. pulcherrimus* and the MRCA of *S. intermedius*, *S. pallidus*, *S. fragilis*, and *S. droebachiensis*. The results of the phylogenetic network analyses underscore the importance of sampling all species of the focal genus or family when testing for introgression. By only sampling a subset of the taxa, introgression may be incorrectly attributed to extant taxa in cases where it occurred on internal branches of the phylogeny. If introgression did occur on an internal branch, there should be considerable overlap in the location of introgressed DNA in each species descendent from that branch.

There are several limitations in the approaches we used to test for introgression. First, it is difficult to quantify the proportion of the genome that is introgressed in each scenario without polymorphism data or populations that are known a priori to have not experienced introgression. However, the *D*_p_ statistic and the PhyloNet reticulation edge weights provide reasonable estimates. Second, the geographic history of speciation, hybridization, and introgression is challenging to interpret given the old divergence times of this group, its limited fossil record, and the fact that the current ranges of the extant taxa may not be representative of their past distributions. This limitation applies to many other marine invertebrate clades due to limitations in the fossil record and shifting ranges due to cycles of sea level rise and fall (Palumbi, 2009). Furthermore, the geographic pattern of hybridization and introgression may be especially complex for marine organisms with high dispersal potential because hybrid zones are more ambiguous.

Our study adds further representation of marine invertebrates to the rapidly growing evidence for hybridization and introgression and will facilitate investigations into how patterns of introgression vary across different organismal groups. Introgression has long been recognized as a significant evolutionary force in plants (Anderson & Hubricht, 1938; Anderson & Stebbins, 1954) but was only recently appreciated in animals (Hedrick, 2013). Historically, it was thought that introgression between marine taxa was rare (Arnold & Fogarty, 2009) and had not occurred among sea urchins (Lessios, 2007). However, reticulate evolution in marine systems may be as common as that of non‐marine taxa (Gardner, 1997), but the difficulty in collecting and observing marine organisms has limited its detection (Arnold & Fogarty, 2009). Although hybridization has been detected in at least five genera of sea urchins (*Diadema*: Lessios & Pearse, 1996, *Lytechinus*: Zigler & Lessios, 2004, *Strongylocentrotus*: Addison & Pogson, 2009, *Pseudoboletia*: Zigler et al., 2012, *Arbaci*a: Lessios et al., 2012), this is the first study that has tested for introgression among sea urchins using genome‐scale data. Among other broadcast spawners, introgression has been detected in *Acropora* corals (Mao et al., 2018), *Mytilus* mussels (Fraïsse et al., 2016; Popovic et al., 2021; Saarman & Pogson, 2015; Simon et al., 2021; Vendrami et al., 2020), *Ophioderma* brittle stars (Weber et al., 2019), *Asterias* sea stars (Harper & Hart, 2007), Western Pacific *Haliotis* abalones (Hirase et al., 2021), and *Ciona* sea squirts (Nydam et al., 2017; Nydam & Harrison, 2011).

### 4.2 On the relative importance of gametic isolation

It is currently believed that the rapid evolution of gamete recognition proteins (GRPs) is a major contributor to reproductive isolation among broadcast spawners. Although reproductive proteins evolve rapidly under positive selection in a wide variety of taxa (Swanson & Vacquier, 2002b), it remains unclear how often this rapid evolution establishes reproductive isolation and causes speciation (Turner & Hoekstra, 2008). Among sea urchins, gametic compatibility can sometimes be maintained for up to five million years and is rarely a bi‐directional barrier to hybridization (McCartney & Lessios, 2004; Zigler et al., 2005). Asymmetric gametic incompatibilities may be the rule rather than the exception (Zigler et al., 2005) and are incapable of preventing gene flow between incipient species (Addison & Pogson, 2009; Lessios, 2011; McCartney & Lessios, 2004), suggesting the importance of additional barriers. Furthermore, bindin is not one of the fastest‐evolving sea urchin genes and only shows evidence of positive selection in three of the seven sea urchin genera studied to date (Geyer et al., 2020). The drivers of selection at bindin are poorly understood and vary across the three genera showing positive selection (*Echinometra*: Metz & Palumbi, 1996; Geyer & Palumbi, 2003; McCartney & Lessios, 2004, *Heliocidaris*: Zigler et al., 2003, *Strongylocentrotus*: Biermann, 1998; Pujolar & Pogson, 2011). In some cases, the selective agent appears to be reinforcement, while in others, it is not clear that the selection at bindin has established sufficient reproductive isolation for the formation of new species.

Within *Strongylocentrotidae*, gametic compatibility *between species* is likely determined by variation in the selective pressures acting on gamete traits *within species* because intraspecific density‐dependent selection acting on gamete traits to maximize fecundity and limit polyspermy also influences susceptibility to heterospecific fertilization (Levitan, 2002a, 2002b; Levitan et al., 2007). Species that more commonly experience sperm‐limiting conditions are selected for high fertilization rates and produce eggs that are more readily fertilized by both conspecific and heterospecific sperm. Conversely, species with higher population densities and high sperm availability likely evolve under sexual conflict and produce faster, more competitive sperm and more sperm‐resistant eggs. This density‐dependent selection has likely led to the asymmetric gametic incompatibilities observed between *S. droebachiensis* and other congeners (Hagström & Lönning, 1967; Levitan, 2002b; Strathmann, 1981) and may have also resulted in asymmetric introgression (Addison & Pogson, 2009). Under the scenario of density‐dependent selection on sperm and egg traits, reproductive isolation between populations should only be strengthened in times or locations of high spawning density. When spawning density is low and populations experience sperm limitation, purifying selection to maximize mating opportunities should favor more easily fertilized eggs and prevent the divergence of GRPs.

Field experiments on *S. droebachiensis* in the Barkley Sound have demonstrated that gametic isolation is not an effective barrier to hybrid matings when spawning females are closer to heterospecific males than conspecific males (Levitan, 2002b). Hybrid fertilizations readily occur when *S. droebachiensis* eggs are swamped by heterospecific sperm, suggesting that some spatial or temporal isolation during spawning is required to prevent hybridization. Work in other broadcast spawner groups has shown that reproductive isolation can evolve without gamete recognition barriers. For example, ecological divergence evolved before GRP divergence in the Western Pacific abalones and maintains species barriers despite ongoing hybridization and introgression (Hirase et al., 2021). In another case, strong reproductive isolation has evolved between the Australian sea urchin species *Pseudoboletia indiana* and *P. maculata* despite only a single amino acid substitution at bindin (Zigler et al., 2012).

The extensive introgression observed among the strongylocentrotid urchins and the lack of a significantly negative correlation between introgression signal and phylogenetic distance, bindin distance, or EBR1 distance indicates that gametic incompatibilities either were not strong enough to prevent significant introgression or evolved after significant introgression had already occurred. Both scenarios are inconsistent with gametic isolation commonly establishing reproductive isolation and causing speciation, suggesting that the GRPs bindin and EBR1 are not speciation genes in the strongylocentrotid family. Other isolating barriers were likely in place and should be investigated further to understand the genetic basis of speciation in strongylocentrotid urchins and other broadcast spawners. Lessios (2007) reviewed isolating barriers in sea urchins and concluded that each prezygotic barrier alone appeared incapable of preventing gene flow between sympatric species. Unfortunately, the relative strength of different isolating barriers has rarely been quantified in pairs of sea urchin sister taxa (Palumbi, 2009).

### 4.3 Possible alternative isolating mechanisms

#### 4.3.1 Postzygotic isolation

How does speciation proceed in high gene flow marine invertebrates with minimal population structure and ecological divergence when geographic barriers are seemingly limited? One possibility is that some postzygotic isolation evolves in allopatry before the evolution of gametic isolation. There are well‐documented cases of hybrid sterility and inviability in interspecific crosses of strongylocentrotid urchins. For example, the *M. nudus* ♀ × *S. intermedius* ♂ cross is lethal (Ding et al., 2007). Although the reciprocal cross produces viable offspring, hybrid larval survival, metamorphosis rates, and juvenile survival are significantly lower than conspecific controls. Furthermore, the surviving juveniles produce very few or no mature gamete cells, a pattern also observed in the *Hemicentrotus pulcherrimus* ♀ × *S. intermedius* ♂ cross (Liu et al., 2020).

In crosses of *S. droebachiensis* × *S. pallidus*, Hagström and Lönning (1967) found that chromosomal abnormalities were frequent during mitosis in embryos of F1 hybrids. Strathmann (1981) performed 10 separate reciprocal crosses between *S. droebachiensis* and *S. pallidus*, but only four hybrids survived to the three‐year mark when spawning was induced, and all were female. The female hybrids were successfully backcrossed in both directions, although backcross fertilization success was much higher with *S. pallidus* males than with *S. droebachiensis* males. Reduced survival of hybrid juveniles has also been found in crosses of female *S. droebachiensis* with male *S. purpuratus* and *M. franciscanus* (Levitan, 2002b) and the cross between *S. purpuratus* and *M. franciscanus* (Newman, 1923). Postzygotic isolation may be even stronger than these studies suggest because intrinsic postzygotic isolation may not appear until generations beyond the F1 if the alleles that cause intrinsic postzygotic isolation are partially recessive in hybrids (Coyne & Orr, 2004). Reproductive barriers may also result from extrinsic (i.e., ecological) postzygotic isolation produced by a mismatch between hybrid individuals and their environment.

#### 4.3.2 Chemical barriers and carbohydrate‐based gamete recognition

The possibility that chemical barriers contribute to reproductive isolation has received limited attention. The egg jelly of broadcast spawners often serves as a chemoattractant to guide conspecific sperm towards the egg, a process called sperm chemotaxis. Conspecific chemoattractant preference has been demonstrated in the abalone species *H. rufescens* and *H. fulgens* (Riffell et al., 2004), although the interaction of gamete recognition proteins is a better predictor of fertilization success in these species (Evans & Sherman, 2013). Sperm chemotaxis has also been described in the sea urchins *Arbacia puctulata* (Ward et al., 1985), *Lytechinus pictus* (Guerrero et al., 2010), and *S. purpuratus* (Ramírez‐Gómez et al., 2020).

In sea urchin fertilization, the acrosome reaction is a precondition for the binding of sperm to the egg and may also be species‐specific in some cases. Alves et al. (1997) found that sulfated polysaccharides in the egg jelly induce the acrosome reaction in a conspecific manner, although the three species tested were quite divergent (*Echinometra lucunter*, *Arbacia lixula*, and *Lytechinus variegatus*). Biermann et al. (2004) similarly found that the jelly coat of *S. droebachiensis* eggs only induces the acrosome reaction in conspecific sperm due to the rapid evolutionary change in the *S. droebachiensis* egg‐jelly fucan. Furthermore, *S. droebachiensis* sperm react with *S. pallidus* and *S. purpuratus* eggs at considerably lower rates than with conspecific eggs. However, the acrosome reaction is not species‐specific between *S. purpuratus*, *M. franciscanus*, and *S. pallidus* (Biermann et al., 2004) or between *Echinometra mathaei* and *Echinometra oblonga* (Metz et al., 1994).

#### 4.3.3 Habitat and temporal isolation

While differences in habitat preference or spawning time could prevent most heterospecific gamete encounters, sea urchin species' ranges commonly overlap, and it is believed that the cues of spawning cycles are too spatially or temporally variable for spawning asynchrony to be an effective barrier (Lessios, 2007). However, species often show depth zonation in areas of range overlap (Lessios, 2007), and slight differences in the timing and location of gamete release among congeners could prevent heterospecific fertilization as sperm rapidly age, disperse, and become diluted following release (Levitan, 1993; Levitan et al., 2004; Pennington, 1985). A short gap in peak spawning times is an effective reproductive barrier for a pair of Panamanian *Montastraea* reef‐building corals (Knowlton et al., 1997) and a pair of Australian subspecies of *Heliocidaris erythrogramma* (Binks et al., 2012). Furthermore, genetic differences in habitat preference were shown to isolate two *Mytilus* mussel species in a contact zone in southern France (Bierne et al., 2003).

## 5 CONCLUSIONS

Although gametic incompatibilities may help maintain species boundaries in strongylocentrotid urchins, gametic isolation does not appear to have been an effective barrier to introgression. The long persistence of gametic compatibility between divergent taxa and evidence of extensive introgression within the family are inconsistent with the rapid evolution of gametic isolation being an important mode of speciation in this family. Additional isolating barriers likely evolved earlier and were more critical in establishing reproductive isolation. The continued divergence of the strongylocentrotid species in the face of significant introgression emphasizes the importance of postzygotic isolation in maintaining species integrities.

## AUTHOR CONTRIBUTIONS

**Matthew R. Glasenapp:** Conceptualization (equal); formal analysis (lead); investigation (lead); methodology (lead); visualization (lead); writing – original draft (lead); writing – review and editing (lead). **Grant H. Pogson:** Conceptualization (equal); funding acquisition (lead); writing – review and editing (supporting).

## FUNDING INFORMATION

Funding for data collection for the study was provided by the National Science Foundation (DEB‐1011061), the STEPS Foundation, Friends of Long Marine Lab, and the Myers Trust. The funding bodies did not participate in research design, sample collection, data analysis, or manuscript writing.

## CONFLICT OF INTEREST STATEMENT

The authors have no conflicts of interest to declare.

### OPEN RESEARCH BADGES

This article has earned an Open Data badge for making publicly available the digitally‐shareable data necessary to reproduce the reported results. The data is available at [https://doi.org/10.7291/D1BT34].

## Supporting information


Appendix S1

Table S1‐S10

## Data Availability

The data and code that support the findings of this study are available on Dryad (https://doi.org/10.7291/D1BT34). Raw sequence reads are available in the NCBI SRA (BioProject PRJNA391452).
