# Supplementary material for: Extensive introgression among strongylocentrotid sea urchins revealed by phylogenomics
Source: Ecol Evol. 2023 Aug 25;13(8):e10446. doi: 10.1002/ece3.10446 (PMC10451471; doi:10.1002/ece3.10446)
Supplement: Supplementary file 1 — Appendix S1 [file ECE3-13-e10446-s002.docx]

Introgression by Phylogenetic Distance

Matthew Glasenapp 2023-07-25

**R Markdown**

1. **Load and display raw data**

data = read.csv("d_k2p.csv") knitr::kable(data, format="markdown")

| taxa_pair | mean_sco_k2p | mean_D | bindin_K2P | ebr1_K2P | mean_dp |
| --- | --- | --- | --- | --- | --- |
| Pdep-Mfra | 0.0247 | 0.0758741 | 0.03947 | 0.05236 | 0.0400867 |
| Spal-Sdro | 0.0155 | 0.0246023 | 0.02024 | 0.04990 | 0.0130619 |
| Sint-Sdro | 0.0152 | 0.0006340 | 0.03350 | 0.04990 | 0.0002934 |
| Sint-Spal | 0.0157 | 0.0112995 | 0.03433 | 0.02757 | 0.0068015 |
| Spur-Sfra | 0.0203 | 0.0848222 | 0.02770 | 0.04779 | 0.0476096 |
| Spur-Sdro | 0.0192 | 0.0511203 | 0.03265 | 0.05358 | 0.0298874 |
| Spur-Spal | 0.0175 | 0.0077695 | 0.02684 | 0.04733 | 0.0047049 |
| Hpul-Sfra | 0.0320 | 0.0126418 | 0.07774 | 0.09618 | 0.0053087 |
| Hpul-Sdro | 0.0314 | 0.0113541 | 0.07672 | 0.08236 | 0.0043718 |
| Hpul-Spal | 0.0298 | 0.0096691 | 0.07315 | 0.09650 | 0.0041781 |
| Hpul-Sint | 0.0312 | 0.0121599 | 0.06793 | 0.09614 | 0.0052346 |

**taxa_pair**: Taxa pairs tested for introgression using Patterson’s *D* statistic. Only taxa pairs are included for tests where *D* was greater than zero in the rooted triplet (((P1,P2),P3),O), where P2 and P3 represent taxa_pair.

**mean_sco_k2p**: Mean Kimura 2-parameter distance across the 6,520 single-copy orthologs inferred by Kober and Pogson (2017)

**mean_D**: Mean Patterson’s *D* statistic (Durand et al., 2011; Green et al., 2010) across tests between taxa_pair where *D* was greater than 0. Patterson’s *D* estimates the excess of derived biallelic site patterns shared between P3 and P2 relative to P3 and P1. The *D* values were taken from Table 2 of Glasenapp and Pogson (2023). The taxa triplets were arranged so that *D* is positive in all cases.

**bindin_K2P**: Bindin Kimura 2-parameter distance between taxa_pair

**ebr1_K2P**: EBR1 Kimura 2-parameter distance between taxa_pair

**mean_dp**: Mean *D*_p_ statistic (Hamlin et al., 2020) across tests between taxa_pair where *D* was greater than 0. *D*_p_ estimates the net proportion of the genome originating from a history of introgression (Hamlin et al., 2020).

1. **Mean Patterson’s *D* x Kimura 2-parameter (K2P) distance**


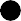


0.08


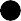
0.06

**Mean Patterson's D Statistic**


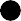


0.04


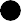


0.02


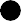

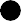


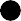

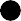

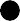
0.00

0.015 0.020 0.025 0.030

# Phylogenetic Distance (K2P Means)

Figure 1: Mean Patterson’s D by phylogenetic distance.

## 2a. Linear regression output for mean Patterson’s *D* by mean K2P distance

lm <- lm(mean_D ~ mean_sco_k2p, data = data) output <- coef(summary(lm)) knitr::kable(output)

|  | Estimate | Std. Error | t value | Pr(>\|t\|) |
| --- | --- | --- | --- | --- |
| (Intercept) | 0.0377800 | 0.0331819 | 1.138571 | 0.284281 |
| mean_sco_k2p | -0.4500302 | 1.3880353 | -0.324221 | 0.753179 |

## Multiple R-squared: 0.01154507

1. **Mean Patterson’s *D* x bindin K2P distance**


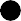


0.08


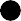
0.06

**Mean Patterson's D Statistic**


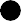


0.04


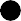


0.02


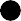

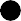

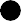

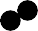

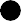


0.00


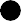
0.025 0.050 0.075

# Bindin Distance (K2P)

Figure 2: Mean Patterson’s D by bindin distance (Kimura 2-parameter).

**3a. Linear regression output for mean Patterson’s *D* by bindin K2P distance**

| lm2 = lm(mean_D ~ bindin_K2P, output2 <- coef(summary(lm2)) knitr::kable(output2) | data = data) |  | | |
| --- | --- | --- | --- | --- |
|  |  |  |  |  |
|  | Estimate | Std. Error | t value | Pr(>\|t\|) |
| (Intercept) | 0.0503436 | 0.0205841 | 2.445757 | 0.0370134 |
| bindin_K2P | -0.4935282 | 0.4028639 | -1.225049 | 0.2516502 |

1. **Mean Patterson’s *D* x EBR1 K2P distance**


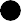


0.08


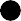
0.06

**Mean Patterson's D Statistic**


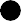


0.04


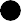


0.02


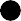

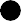


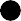


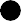
0.00

0.04 0.06 0.08

# EBR1 Distance (K2P)

Figure 3: Mean Patterson’s D by EBR1 distance (Kimura 2-parameter).

**4a. Linear regression output for mean Patterson’s *D* by EBR1 K2P distance**

lm3 = lm(mean_D ~ ebr1_K2P, data = data) output3 <- coef(summary(lm3)) knitr::kable(output3)

| Estimate | Std. Error | t value | Pr(>\|t\|) |
| --- | --- | --- | --- |
| (Intercept) 0.0513891 | 0.0257329 | 1.9970231 | 0.0769211 |
| ebr1_K2P -0.3764003 | 0.3799159 | -0.9907464 | 0.3476966 |

1. **Mean *D*_p_ x phylogenetic, bindin, and EBR1 distances**

0.04

0.04

0.04

0.03

**Mean DP**

0.03

0.03

0.02

0.02

0.02

0.01

**Mean DP**

**Mean DP**

0.01

0.01

0.00

0.015 0.020 0.025 0.030

**Phylogenetic Distance (K2P Means)**

0.00

0.025 0.050 0.075

**Bindin Distance (K2P)**

0.00

0.04 0.06 0.08

**EBR1 Distance (K2P)**

Figure 4: Mean Dp by phylogenetic distance, bindin distance, and EBR1 distance.

1. ***Strongylocentrotus* genus only**

*# Extract Strongylocentrotus comparisons only* strongylocentrotus = data[2:7,] knitr::kable(strongylocentrotus, format="markdown")

|  | taxa_pair | mean_sco_k2p | mean_D | bindin_K2P | ebr1_K2P | mean_dp |
| --- | --- | --- | --- | --- | --- | --- |
| 2 | Spal-Sdro | 0.0155 | 0.0246023 | 0.02024 | 0.04990 | 0.0130619 |
| 3 | Sint-Sdro | 0.0152 | 0.0006340 | 0.03350 | 0.04990 | 0.0002934 |
| 4 | Sint-Spal | 0.0157 | 0.0112995 | 0.03433 | 0.02757 | 0.0068015 |
| 5 | Spur-Sfra | 0.0203 | 0.0848222 | 0.02770 | 0.04779 | 0.0476096 |
| 6 | Spur-Sdro | 0.0192 | 0.0511203 | 0.03265 | 0.05358 | 0.0298874 |
| 7 | Spur-Spal | 0.0175 | 0.0077695 | 0.02684 | 0.04733 | 0.0047049 |

## 6a. Mean Patterson’s *D* x phylogenetic, bindin, and EBR1 distances

0.08

**Mean Patterson's D Statistic**

0.08

0.06

**Mean Patterson's D Statistic**

0.08

0.06

**Mean Patterson's D Statistic**

0.04

0.04

0.04

0.00

0.02

0.02

0.015 0.016 0.017 0.018 0.019 0.020

**Phylogenetic Distance (K2P Means)**

0.00

0.021 0.027 0.033

**Bindin Distance (K2P)**

0.00

0.030 0.035 0.040 0.045 0.050

**EBR1 Distance (K2P)**

Figure 5: Mean Dp by phylogenetic distance, bindin distance, and EBR1 distance.

## 6b. Linear regression output for mean Patterson’s *D* by bindin K2P distance

lm4 = lm(mean_D ~ mean_sco_k2p, data = strongylocentrotus) output4 <- coef(summary(lm4))

knitr::kable(output4)

|  | Estimate | Std. Error t value | Pr(>\|t\|) |
| --- | --- | --- | --- |
| (Intercept) | -0.1974922 | 0.0630373 -3.132942 | 0.0350855 |
| mean_sco_k2p | 13.2031021 | 3.6346563 3.632559 | 0.0221097 |

## Multiple R-squared: 0.7673808

## 6c. Mean *D*_p_ x phylogenetic, bindin, and EBR1 distances

0.06

0.04

**Mean Dp**

0.02

0.04

0.03

**Mean Dp**

0.02

0.04

0.03

**Mean Dp**

0.02

0.00

0.01

0.01

0.015 0.016 0.017 0.018 0.019 0.020

**Phylogenetic Distance (K2P Means)**

0.00

0.021 0.027 0.033

**Bindin Distance (K2P)**

0.00

0.030 0.035 0.040 0.045 0.050

**EBR1 Distance (K2P)**

Figure 6: Mean Dp by phylogenetic distance, bindin distance, and EBR1 distance.

## 6d. Linear regression output for mean *D*_p_ x mean K2P distance

lm5 = lm(mean_dp ~ mean_sco_k2p, data = strongylocentrotus) output5 <- coef(summary(lm5))

knitr::kable(output5)

Estimate Std. Error t value Pr(>|t|)

(Intercept) -0.1130355 0.0340277 -3.321869 0.0293273

mean_sco_k2p 7.5490507 1.9619971 3.847636 0.0183396

## Multiple R-squared: 0.7872827

1. **References**

Durand, E. Y., Patterson, N., Reich, D., & Slatkin, M. (2011). Testing for ancient admixture between closely related populations. Molecular Biology and Evolution, 28(8), 2239–2252. [https://doi.org/10.1093/molbev/](https://doi.org/10.1093/molbev/msr048) [msr048](https://doi.org/10.1093/molbev/msr048)

Green, R. E., Krause, J., Briggs, A. W., Maricic, T., Stenzel, U., Kircher, M., Patterson, N., Li, H., Zhai, W., Fritz, M. H.-Y., Hansen, N. F., Durand, E. Y., Malaspinas, A.-S., Jensen, J. D., Marques-Bonet, T., Alkan, C., Prüfer, K., Meyer, M., Burbano, H. A., . . . Pääbo, S. (2010). A draft sequence of the neandertal genome. Science, 328(5979), 710–722. <https://doi.org/10.1126/science.1188021>

Hamlin, J. A. P., Hibbins, M. S., & Moyle, L. C. (2020). Assessing biological factors affecting postspeciation introgression. Evolution Letters, 4(2), 137–154. <https://doi.org/10.1002/evl3.159>

Kober, K. M., & Pogson, G. H. (2017). Genome-wide signals of positive selection in strongylocentrotid sea urchins. BMC Genomics, 18(1), 555. <https://doi.org/10.1186/s12864-017-3944-7>
